# Exploring the relationship between seasonal changes and bipolar disorder relapses

**DOI:** 10.1192/j.eurpsy.2025.1049

**Published:** 2025-08-26

**Authors:** A. Nunes, M. Almeida e Silva, J. Fonseca Barbosa, J. Fernandes Fontes, M. Andrade

**Affiliations:** 1 Psychiatry, Hospital Júlio de Matos - ULS São José, Lisbon, Portugal

## Abstract

**Introduction:**

Bipolar disorder (BD) is a mental health disorder characterized by episodes of mania or hypomania alternating with depression, and it is known that seasonal changes can have an impact on the risk of relapse. Circadian rhythm - which works as an internal biological clock that regulates sleep-wake cycles, hormone production and mood stability -, plays a crucial role in the course of the disease, and it is likely that it influences relapse during seasonal changes, through mechanisms not entirely understood.

**Objectives:**

Review the relationship between seasonal changes and bipolar disorder relapse, focusing on circadian rhythm disruption, including possible pathophysiological pathways and treatment options.

**Methods:**

Narrative review of articles published on Pubmed’s database using the following keywords and their combinations: *bipolar disorder*, *circadian rhythms*, *seasonal* and *sleep disturbances*, screening for relevance.

**Results:**

Several studies show us that manic episodes are associated with transition into spring and summer and depressive ones with transition into autumn and winter. Seasonal changes result in alterations in daylight exposure, which in turn, through the cardinal role of the suprachiasmatic nuclei in the hypothalamus, lead to disruptions in sleep-wake cycles, impacting melatonin and cortisol levels, which can contribute to mood instability. These hormones are also subjected to changes by other shifts in biological rhythms such as body temperature regulation, that come with seasonal transitions. On the other hand, light exposure also influences neurotransmission, particularly of serotonin and dopamine, with consequences on energy, mood and reward processing and arousal. There might also be a role for genetic polymorphisms like CLOCK, BMAL1 and PER, that influence sleep patterns and hormonal regulation, and therefore can predispose some people to mood disorders. Furthermore, there are important social factors related to seasonal changes, such as increases or decreases in social activities, that can impact mood. Therapeutic approaches that target circadian rhythm, such as light therapy and chronotherapy (including options like sleep deprivation and phase advance therapies), can be useful in decreasing relapse episodes. Additionally, simple psychoeducation on the matter, regarding maintenance of regular sleep schedules and social activities, might be helpful in preventing or, at the very least, decreasing relapses.

**Conclusions:**

Seasonal changes play a relevant role in both manic and depressive relapses in BD through their role in circadian rhythm disruption, by way of a myriad of mechanisms. Future investigation should focus on these mechanisms and others that might possibly be involved, allowing us to reach more targeted treatment and even preventative measures to diminish relapse episodes in BD.

**Disclosure of Interest:**

None Declared

